# Potential use of corneal confocal microscopy in the diagnosis of Parkinson’s disease associated neuropathy

**DOI:** 10.1186/s40035-020-00204-3

**Published:** 2020-07-02

**Authors:** Ning-Ning Che, Hong-Qi Yang

**Affiliations:** 1 Department of Neurology, Henan Provincial People’s Hospital, School of Clinical Medicine, Henan University, Zhengzhou, 450003 China; 2 Department of Neurology, People’s Hospital of Zhengzhou University, School of Clinical Medicine, Zhengzhou University, Zhengzhou, 450003 China

**Keywords:** Parkinson’s disease, Peripheral neuropathy, Corneal confocal microscopy, Small fiber neuropathy

## Abstract

Parkinson’s disease (PD) is a chronic, progressive neurodegenerative disease affecting about 2–3% of population above the age of 65. In recent years, Parkinson’s research has mainly focused on motor and non-motor symptoms while there are limited studies on neurodegeneration which is associated with balance problems and increased incidence of falls. Corneal confocal microscopy (CCM) is a real-time, non-invasive, in vivo ophthalmic imaging technique for quantifying nerve damage in peripheral neuropathies and central neurodegenerative disorders. CCM has shown significantly lower corneal nerve fiber density (CNFD) in patients with PD compared to healthy controls. Reduced CNFD is associated with decreased intraepidermal nerve fiber density in PD. This review provides an overview of the ability of CCM to detect nerve damage associated with PD.

## Background

Parkinson’s disease (PD) is a chronic, progressive neurodegenerative disease affecting about 2 ~ 3% of population above the age of 65, and is the second largest neurodegenerative disease after Alzheimer’s disease [1]. The cardinal manifestations of motor symptoms of PD are bradykinesia, rest tremor, muscle stiffness, and posture instability and gait disorder, while cognitive impairment, psychiatric presentation, gastrointestinal symptom, autonomic dysfunction, hyposmia, sleep problems are the mainstay of non-motor symptoms [2–4]. Studies have been focusing on motor symptoms and non-motor symptoms in PD [5]. There are limited studies on peripheral neuropathy driven by PD [6–8]. A study showed that polyneuropathy is more frequent in PD patients than in age-matched controls [9]. Although peripheral neuropathy is a frequently underestimated clinical symptom in PD, the estimated overall incidence is 19 to 55% [9, 10]. Age is a risk factor for the development of peripheral neuropathy in PD patients. The risk of peripheral neuropathy increased by approximately 8% with each year of aging [11]. Study showed that peripheral neuropathy increases the chance of fall, affect balance and gait, thus seriously affecting the quality of life in affected PD patients [12]. In a recent study, the frequency of falls almost tripled in PD patients with neuropathy as compared with PD patients without neuropathy [13]. On the other hand, the symptoms of peripheral neuropathy may overlap with numbness and muscle spasm which developed at the distal end of the extremity in late course, so it can be easily neglected by the treating physicians. This in turn may aggravate the clinical symptoms and further affect the quality of life.

The etiology and pathogenesis of peripheral neuropathy in PD is still controversial. Some researchers suggest that peripheral neuropathy is related to the metabolic pathway of levodopa [10]. The notion comes from studies which showed that levodopa can induce peripheral nerve injury by mediating homocysteine and vitamin B12 circulation [10, 11, 14]. For instance, a research by Ceravolo et al. showed increased prevalence of neuropathy in PD patients, and the duration of exposure to levodopa along with age was the main risk factor for the development of neuropathy [11]. At the same time, a large number of studies also indicated that, as a neurodegenerative disease, PD can also affect the peripheral nervous system [15–18]. For example, phosphorylated α-synuclein has been found to deposit in heart sympathetic nerve, colon nerve, glossopharyngeal nerve, vagus nerve and other peripheral nervous systems [15–17]. Study by Mu et al. showed that α-synuclein aggregates were identified in the pharyngeal sensory nerves of pathologically confirmed PD patients but not controls. And these PD patients with swallow problems had more α-synuclein density than those without dysphagia [16]. At the same time, skin biopsy also confirmed that deposition of phosphorylated α-synuclein in the cutaneous nerve fibers, which may result in damage of small nerve fibers [18]. These findings might suggest that peripheral neuropathy involvement was just part of the PD clinical symptomatic spectrum and might be directly affected by pathologic processes of PD. Using clinical scoring system and nerve conduction studies (NCS), the clinical evaluation and neurophysiological measurements of peripheral neuropathy in PD patients was carried out simultaneously [19]. While the characteristics and discrepancy between the number of patients with clinical and NCS detected peripheral neuropathy differ significantly. Therefore, researchers suggested small fiber neuropathy may be intrinsic to PD pathogenesis in the early stage of PD and large fiber neuropathy may be complicated by levodopa therapy, usually in advanced PD patients [20].

At present, the diagnosis of peripheral neuropathy depends mainly on neuropathy-related symptoms, signs and electrophysiological techniques. But the variability of clinical symptoms and signs is large and the repeatability is poor. NCS is considered to be a reliable method for the diagnosis of peripheral neuropathy, but it can only evaluate large, myelinated nerve fibers. However, the ability to assess the damage of small nerve fibers, especially after therapeutic intervention, is limited [21]. Quantitative sensory testing (QST) is non-invasive, easy to be carried out and has good repeatability, but its shortcoming is that the subjective coordination of patients has a greater impact on the final results [22]. The intraepidermal nerve fiber density (IENFD) is considered as the most objective index for the diagnosis and quantification of small fiber neuropathy, but the invasive character limited its clinical application [23]. Therefore, there is an urgent need for a non-invasive, stable and sensitive detection method for the diagnosis of small fiber neuropathy in Parkinson’s disease.

Corneal confocal microscopy (CCM) is an ophthalmic imaging technique for quantifying nerve morphology in peripheral neuropathy and central neurodegenerative disorders [6, 7, 24, 25]. CCM can be used to detect and evaluate the progress of systemic diseases with peripheral neuropathy in the early stage of disease, such as diabetic peripheral neuropathy and other small fiber neuropathy [24, 26–29]. A study found corneal nerve fiber length was also inversely correlated to glycated hemoglobin and duration of diabetes [30], which suggests that CCM may play an important role in evaluating pathogenetic treatments. Studies have reported corneal nerve pathology associated with PD [6–8, 31]. In this review, the significant advance made to date in this field by the use of this in vivo ophthalmic imaging technique was summarized. The review provides an overview of the ability of CCM to detect nerve damage associated with PD.

## Main text

### Corneal confocal microscopy (CCM)

At present, CCM is widely used in clinical diagnosis and therapeutic effect monitoring of ocular diseases and systemic diseases. In clinical practice, the illumination source utilized in CCM (Heidelberg Retinal Tomograph III with Rostock Cornea Module) is a 670 nm wavelength helium neon diode laser. This is a class I laser and does not pose any ocular safety hazard. The laser beam spot was 1 μm in diameter and the instrument field of view was 400 × 400 μm with a 63× objective lens.

Before CCM examination, the camera (objective lens tip) is prepared in advance. Lidocaine was used to anesthetize each eye and then the subject is seated comfortably and instructed to fixate on an outer fixation light. The CCD camera was used to correctly position the applanating cap onto the cornea. Images from the central corneal area at sub-basal plexus were obtained and captured using the “Section” mode by an experienced examiner. CCM images of the best quality were analyzed using validated, semi-automated software. The protocol has been described in details by Malik et al. previously [32]. The corneal nerve morphology can be quantified using an automated software [33]. Study demonstrated automatic analysis had the same ability with the manual analysis which has previously demonstrated encouraging clinical performance for the stratification of neuropathic severity [33]. Studies by Petropoulos et al. and Ostrovski et al. suggested automated software and manual software also had high correlations [34, 35]. The initial step in automated software analysis is CCM image enhancement and nerve fiber detection. Second step is quantification of the three morphometric parameters. CCM has been validated for the diagnosis of diabetic neuropathy by quantifying the central cornea nerve morphology. Petropoulos et al. has reported nerve fiber loss in the Inferior whorl (IW) and recommended to quantify the corneal nerve morphology in central cornea and in the IW [36]. IW length can even detect the abnormality even in patients without diabetic peripheral neuropathy [36]. The study by Kalteniece et al. showed that there is more prominent distal corneal nerve fiber damage at the IW in patients with diabetic neuropathy [37]. About 30% of patients with a reduction in IW length had a normal corneal nerve fiber length, while only 13.5% of patients with a normal IW length had an abnormal corneal nerve fiber length. Combination of corneal nerve fiber length at the central and IW may increase the sensitivity of CCM in detecting peripheral neuropathy in PD.

### Corneal nerve fibers

**Fig. 1:**
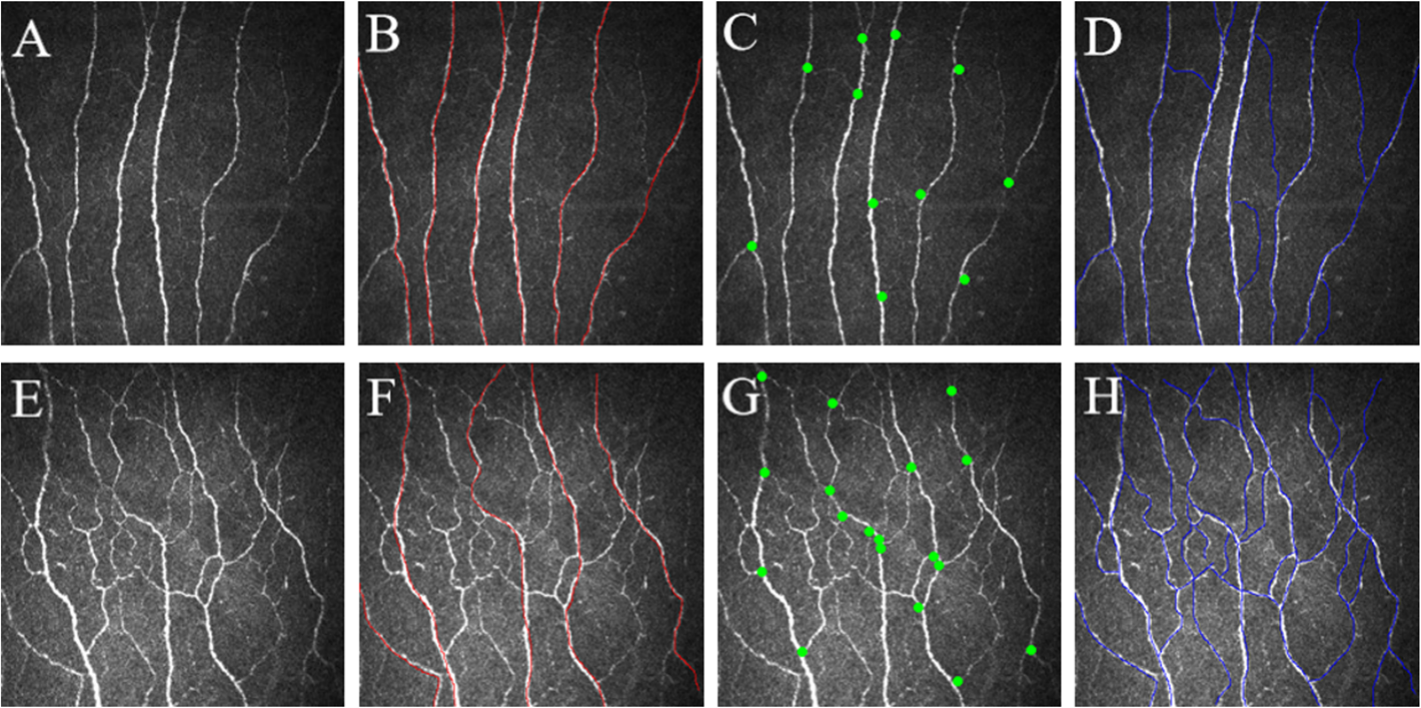
The representative corneal confocal microscopy (CCM) image of corneal nerve fibers in healthy control (**a** ~ **d**) and Parkinson’s disease (**e** ~ **h**) patients. In CCM, the corneal nerve plexus is beaded, linear homogeneous and highly reflective (**a**, **e**). Parkinson’s disease patients showed decreased corneal nerve fiber density (**f**), increased corneal nerve branch density (**g**) and corneal nerve fiber length (**h**) as compared with control group (**b**, **c** and **d** respectively). For a clearer illustration, nerve fiber trunks were highlighted in red line (**b**, **f**), branch origins were represented by the green dots (**c**, **g**) and corneal nerve fiber lengths shown in blue line (**d**, **h**)

Cornea is the most densely innervated area of human tissues with about 7000 free nerve endings per square millimeter [38]. Corneal nerve fibers originate from the ophthalmic branches of the trigeminal nerve and contain A delta nerve fibers and unmyelinated C nerve fibers. Nerve innervation plays an important role in maintaining corneal integrity. Dynamic changes in the distribution and morphology of corneal nerve fibers has been observed in various ocular and systemic diseases state. The transparency character of the cornea makes it possible to observe the morphology of nerve fibers directly and noninvasively. In CCM, the corneal nerve plexus is beaded, linear homogeneous and highly reflective (Fig. 1) (Che N-N, Yang H-Q, Ding G-X, et al.: The study of corneal confocal microscopy in Parkinson’s disease, unpublished). The widely used parameters for evaluating the morphology of corneal nerve fibers are as follows: (a) Corneal nerve fiber density (CNFD): the number of all nerve trunk fibers per square millimeter; (b) Corneal nerve branch density (CNBD): the number of branch nerves originating from the main nerve within a square millimeter; (c) Corneal nerve fiber length (CNFL): the sum of all nerve fibers per square millimeter; and (d) Corneal nerve fiber tortuosity (CNFT): the curvature of the total nerve fibers [39, 40].

### Application of CCM in diabetic peripheral neuropathy and other small fiber neuropathy

As a rapid, non-invasive, ophthalmic imaging technique for quantifying corneal nerve morphology. CCM has been extensively used in the study of diabetic peripheral neuropathy and autonomic neuropathy [37, 41–43]. American diabetes association and the Toronto expert consensus panel on diabetic neuropathy both endorse CCM as a valid clinical technique for measures of small fiber damage and repair in diabetic neuropathy [44, 45]. Measurement of small fiber neuropathy in Fabry disease, idiopathic small fiber neuropathy and Charcote-Mariee-Tooth type 1A patients has also been reported recently [28, 46, 47]. Chen et al. found that CNFD, CNFL, CNBD and IENFD was significantly decreased in diabetic patients with peripheral neuropathy than that of the control group [48]. There was no significant difference in the area of ROC curve between the two methods (CCM and IENFD) in diagnosing peripheral neuropathy [48]. This study further verified that, as an in vivo ophthalmic imaging modality, CCM has the potential to be a non-invasive, objective, ophthalmic imaging biomarker for identifying small fiber damage, and is an ideal alternative method for the early diagnosis of peripheral neuropathy.

### Potential application of corneal confocal microscopy as a biomarker of neurodegeneration in Parkinson’s disease

#### The relationship between CCM parameters and PD clinical profiles

Several studies have found changes of CCM parameter in PD patients. The results from Kass-Iliyya showed decreased CNFD but increased CNBD and CNFL in PD patients as compared with control patients [6]. Our unpublished data in PD patients also observed the similar phenomenon (Fig. 1) (Che N-N, Yang H-Q, Ding G-X, et al.: The study of corneal confocal microscopy in Parkinson’s disease, unpublished). In contrary to these results, Podgorny et al. [7] found PD patients had significantly reduced CNBD and CNFL as compared to controls. Although CNFD also decreased in PD group, the difference didn’t reach statistical significance. Another study by Misra et al. showed that corneal sub-basal nerve plexus density (the total length of all nerves in corneal sub-basal nerve density) decreased by 50% compared with healthy controls [8]. The discrepancy results among various study groups may be related with the clinical profiles of enrollment PD patients. The patients from Podgorny’s study were early untreated PD patients, but the patients from Kass-Iliyya have been treated for some time and disease course has lasted for a period of time [6]. And the patients in Misra’s study were moderate to severe PD patients with Hoehn-Yahr of 3 to 4 stages [8]. Other explanations for these differences maybe due to the compensatory regeneration of branching nerves, which leads to the increase of CNBD and CNFL after the decrease of CNFD. Previous study by Nolano et al. found patients showed not only significant loss of epidermal nerve fibers and Meissner corpuscles, but also presence of vasoactive intestinal peptide-immunoreactive fibers and the increased calcitonin gene related peptide-immunoreactive fibers in the subepidermal plexus, which were proof of nerve regeneration attempts [49]. The presence of increased nerve branching, sprouting and clustering suggest coexistence of degenerative and regenerative processes in Parkinson’s disease [49]. Indeed, in pancreas and kidney transplantation, CNBD was the first measure to show improvement at 6 months and continued to improve significantly at 12 months compared with baseline [21]. In these studies, the increased CNBD is likely to reflect attempted nerve regeneration in the specific course of a disease. The neurodegeneration be balanced or even overtaken by regeneration may imply that peripheral nerve may have neuroplasticity in Parkinson’s disease. The results by Kass-Iliyya also showed that CNBD and CNFL were correlated with UPDRS-III and autonomic dysfunction. But there was no correlation between CCM parameters and disease duration, cumulative levodopa dose or pain score. They concluded that CCM identifies corneal nerve fiber pathology, which correlated with autonomic symptoms, parasympathetic deficits and motor scores in PD patients [6]. Misra et al. showed that decreased corneal sub-basal nerve density was closely related to cognitive impairment, suggesting that corneal nerve changes were the intrinsic characteristics of neurodegenerative diseases rather than being influenced by external factors [8]. The utility of CCM in mild cognitive impairment (MCI) and dementia (including Alzheimer’s disease, vascular dementia and mixed dementia) also showed positive results. Study by Ponirakis et al. found that CNFD, CNBD and CNFL showed a progressive reduction trend in MCI and dementia as compared to age-matched healthy controls [25]. The corneal nerve fiber damage was significantly associated with decline in cognitive function and functional independence in patients with MCI and dementia. But whether CCM may have the same significance in PD with MCI, Parkinson’s disease dementia and dementia with Lewy bodies still need to be further investigated.

Studies have shown that corneal nerve changes were related to autonomic nerve function and cardiac parasympathetic nerve function [6]. Since early autonomic nerve dysfunction was associated with faster disease progression and shorter survival time [50], whether peripheral neuropathy manifested by corneal nerve damage will affect disease progression remains to be investigated. A study by Merola et al. showed that PD patients with polyneuropathy had worse cognitive, axial motor, autonomic, and nonmotor features compared with PD patients without polyneuropathy, which suggests that polyneuropathy may represent an independent peripheral marker of a severe PD phenotype [51]. Furthermore, Nolano et al. measured IENFD of 28 PD patients through 1 to 108 months of follow-up and found that IENFD correlated with disease duration and disease severity. These findings imply that small fiber pathology parallels disease progression in PD [52].

Decreased blink rate is a common finding in PD patients, it might be reasonably assumed that the cause of the decreased blink rate in PD is simply a manifestation of bradykinesia, which mean arising centrally. However, Reddy et al. reported a strong correlation between blink rate and corneal sub-basal nerve density. In four patients with PD, it showed that blink rate may be influenced by the nerve density in the sub-basal layer [31]. But in Misra’s study, there was no statistically significant correlation between corneal sub-basal nerve density and blink rate [8]. Besides small sample size, the distinct design, population characteristics, diagnostic accuracy may explain the results heterogeneity between the two studies. Whether reduced blink rate in PD is correlated with corneal denervation remains to be further studied.

For a better understanding the role of CCM in PD research, related studies and results were summarized in the Table 1.

**Table 1:** Studies investigating corneal confocal microscopy (CCM) in Parkinson’s disease (PD)

Studies	Aims	Study cohort	Main findings	Reference
Kass-Iliyya et al. (2015)	To determine whether CCM can demonstrate small nerve fiber damage in PD. To identify relationships between CNP, IENFD and clinical features of PD.	cross-sectional (52 subjects: 26 PD, 26 controls)	CNFD was reduced but CNBD and CNFL were increased in PD compared to healthy controls. CNBD and CNFL but not CNFD correlated inversely with UPDRS-III and SCOPA-AUT. IENFD is also reduced and correlates with CNFD and motor symptoms.	[6]
Podgorny et al. (2016)	To determine if peripheral neuropathy occurs in early untreated PD.	cross-sectional (48 subjects: 26 PD, 22 controls)	CNFL, CNBD, CNFD were reduced in PD compared to healthy controls, but CNFD didn’t have significantly difference. NCS and IENFD found no significant difference between groups.	[7]
Misra et al. (2017)	To examine the ocular surface in patients with moderately severe PD.	cross-sectional (30 subjects: 15 PD, 15 controls)	Corneal sub-basal nerve plexus density was markedly reduced in patients with PD compared with controls. Sub-basal corneal nerve density was a significant positive correlation between ACE-R scores.	[8]
Reddy et al. (2013)	To examine the ocular surface in patients with PSP and PD.	cross-sectional (16 subjects: 4 PD, 7 PSP, 5 controls)	There were no differences in corneal sub-basal nerve density between the 3 groups.	[31]
Arrigo et al. (2018)	To describe corneal innervation and trigeminal alterations in drug-naive patients with PD.	cross-sectional (15 subjects: 3 PD, 12 controls)	Deep nerve tortuosity and the number of beadings were increased in patients with PD compared with controls.	[53]
Daggumilli et al. (2019)	To evaluate the progression of corneal endothelial changes in patients with PD on long-term oral amantadine therapy.	1-year follow-up (150 subjects: 90 PD with amantadine, 30 PD naïve amantadine, 30 controls)	SBNFLD was decreased in PD amantadine and PD amantadine naive group compared with controls after 1-year follow-up.	[54]

*CCM* corneal confocal microscopy, *PD* Parkinson’s disease, *CNP* corneal nerve parameters, *IENFD* intraepidermal nerve fiber density, *CNFD* corneal nerve fiber density, *CNBD* corneal nerve branch density, *CNFL* corneal nerve fiber length, *UPDRS- III* Unified Parkinson’s Disease Rating Scale III, *SCOPA-AUT* the scale for outcomes in Parkinson’s disease for autonomic symptoms, *NCS* nerve conduction studies, *ACE-R* Addenbrooke’s cognitive examination- revised, *SBNFLD* sub-basal nerve fiber layer density

#### Side-specific relationship between CCM and motor symptoms

When the CCM parameters between the clinically more affected side and clinically less affected side of asymmetry PD patients were compared, Misra et al. found no significant difference in the sub-basal corneal nerve plexus density values between the two sides [8]. This is consistent with the study of Kass-Iliyya [6]. In addition, neither corneal sensitivity (measured by non-contact corneal aesthesiometer) was found to differ significantly between the clinically more affected side and the clinically less affected side. In unilateral affected PD patients, Donadio et al. revealed no difference in phosphorylated α-synuclein between the two sides of cervical skin biopsy [55], suggesting that the degree of neurodegeneration of dopaminergic neurons in substantia nigral/striatum may not be related to the degree of denervation of peripheral nerves and cardiac sympathetic nerve [56]. In Lauria’s study, PD patients and healthy controls underwent skin biopsies at bilateral legs and also underwent follow-up biopsies 20 days later. The study found IENFD did not differ in more affected versus less affected side of PD patients and healthy controls [57]. But Jeziorska et al. found IENFD was lower in more affected versus less affected side in PD patients [58]. However, the study by Nolano et al. showed decreased IENFD in the more affected at baseline, but more nerve fiber loss in the less affected side with longer disease duration [52]. The author proposed the initial asymmetry of IENFD tends to blur along disease course as it occurs with motor impairment. That is, the regeneration may compensate for nerve degeneration in early disease stage, but this regenerative capacity may decline over time as the disease progress. Discrepancies between studies are likely to be attributable to a variety of factors, including differences in case selection, disease stage, biopsy sites, study design, and loss to follow-up. Thus, side-specific relationship between CCM and motor symptoms is complex and need to be investigated in longitudinal studies.

#### Role of CCM in PD early diagnosis

Visual impairment is a common sensory symptom in Parkinson’s disease. It is characterized by blurred vision, diplopia, decreased photosensitivity and hallucination [59]. These symptoms are mostly related to retina and can be present for more than 10 years before motor symptoms development. Thus, the study of retinal changes in PD patients has been used by some researchers to assess disease progression and aid in early diagnosis [60, 61]. One study indicated that PD patients showed prominent alterations in corneal innervations and in trigeminal diffusion MRI parameters. As compared with control group, PD patients have increased deep nerve tortuosity and the number of beadings. MRI diffusion study showed that PD patients also displayed decreased fractional anisotropy and increased mean diffusity as compared with control patients. Corneal innervation changes might occur earlier than retinal ones in PD patients and the author claims that CCM analysis might provide as early biomarkers for better PD evaluation and earlier diagnosis [53]. In Podgorny’s study, neurological examination and NCS were performed in early untreated PD and controls patients and found no significant difference between groups [7]. More sensitive and informative tests including skin biopsy and CCM were performed in two groups. While CNBD and CNFL significantly decreased as compared with control group, no significant change in IENFD was found. These results mean that in the early stage of PD, when no obvious changes can be found by IENFD, notable changes can be detected by CCM [7]. Cornea is the most densely innervated area of human tissue [38], changes in corneal nerve fibers can occur before changes in cutaneous nerve fibers [62], thus CCM may be used as an effective tool for early diagnosis in PD.

#### Potential role of CCM in differential diagnosis of PD from parkinsonism

Although great advances have been made in neuroimaging and genetics, the diagnosis of PD at present remains primarily clinical. Since definite diagnosis can only be obtained pathologically, misdiagnosis is unavoidable. Even the most recently published diagnostic criteria were used, researchers reported diagnostic accuracy of about 80%, with the most frequently misdiagnoses were multiple system atrophy (MSA), Lewy body dementia (LBD), and progressive supranuclear palsy (PSP) [63, 64]. Lack of reliable and easily accessible biomarkers may explain the suboptimal accuracy of the clinical diagnosis. α-synuclein is a major component of Lewy body and Lewy neuritis, and deposition of phosphorylated α-synuclein in cutaneous nerve fibers has been demonstrated in skin biopsies and autopsies of patients with PD. Higher α-synuclein is associated with greater autonomic dysfunction and more advanced PD. In the cervical skin site, researchers found phosphorylated α-synuclein in PD patients but not in Parkinsonism and controls [65]. Similarly, phosphorylated α-synuclein staining was measured in sympathetic skin nerve fibers, correlating with an age-independent denervation of autonomic skin elements, but not in MSA and essential tremor patients [18]. In another study, salivary α-synuclein was decreased with control group, and the change of α-synuclein levels appeared to be negatively correlated with the severity of motor symptoms as measured with UPDRS score [66]. These results rational the use of phosphorylated α-synuclein as a potential biomarker for PD and may help to differentiate PD from other Parkinsonism. But these studies and others all have drawbacks, including difficulty in collecting CSF samples at the same time and possible blood contamination. In addition, the standardization of skin biopsy procedure and biopsy site need to be established before it became a tool for the diagnosis of idiopathic PD. Study by Reddy showed no difference in sub-basal nerve density between 4 PD patients and 7 cases of PSP [31]. Considering the relatively small sample size, and different clinical profiles, the interpretation of these data need caution. Larger sample size and multi-center study is needed to better confirm the role of CCM in the differential diagnosis of PD and Parkinsonism.

#### Potential role of CCM in evaluating the efficacy of drugs in PD

At present, the effect of drug in PD peripheral neuropathy is still unclear. Daggumilli et al. found that corneal sub-basal nerve fiber layer significantly decreased in both PD amantadine and PD amantadine naive group compared with healthy control group at 1-year follow-up. Further, corneal sub-basal nerve fiber layer was lower in PD amantadine compared with PD amantadine naive group [54]. It suggests amantadine may affect corneal nerve fibers. This study also means that CCM may be an attractive non-invasive and reproducible measure to monitor the efficacy of drug in PD intervention. Traditional neuropathy treatments included folate and VitB12. However, the role of these drugs in PD requires evaluation. In a study by Brines et al., the effects of ARA 290 (a nonhematopoietic peptide) on diabetic peripheral neuropathy were analyzed [67]. ARA 290 exhibited an improvement in hemoglobin A1c, lipid profiles and neuropathic symptoms, it also increased in corneal nerve fiber density in subgroup analysis [67]. Although these interventions may have a role in diabetic peripheral neuropathy, whether they will have the same role on neuropathy in Parkinson’s disease still requires further evaluation.

### Application of CCM in other central nervous system disorders

There are currently an increasing number of studies showing corneal nerve abnormalities in typically central neurodegenerative diseases like multiple sclerosis, dementia, Wilson disease, Freidreich’s ataxia, and even cerebral vascular disease [25, 68–75]. For example, significant reduction in CNFD and CNFL were recorded in Friedreich’s ataxia as compared with the controls [75]. CCM parameters correlated with genotype and Friedreich’s ataxia rating scales, suggest CCM quantification of corneal nerve morphology maybe a sensitive ophthalmic imaging biomarker for quantifying the severity of neurologic disease in individuals with Friedreich’s ataxia. Although CCM parameters change in a broad of central nervous system situations, the detailed mechanism is still unclear. Whether CCM changes are the results, or just by-standers of underlying pathogenesis, still need to be further investigated. The related studies and main findings were shown in Table 2.

**Table 2:** Studies investigating corneal confocal microscopy (CCM) in central nervous system disorders

Category	Studies	Aims	Study cohort	Main findings	Reference
MS	Bitirgen et al. (2017)	To assess corneal sub-basal nerve plexus morphologic features, corneal DC density in patients with MS.	cross-sectional (87 subjects: 57 MS, 30 controls)	CNFD、CNBD、CNFL were reduced but DC density was increased in patients with MS compared with healthy controls.	[68]
	Mikolajczak et al. (2017)	To investigate the effect of MS on corneal nerve fibers and DC in the sub-basal nerve plexus using in CCM.	cross-sectional (52 subjects: 26 MS, 26 controls)	significant reduction in total corneal nerve fiber density in MS patients compared to controls. DC density was similar in both groups.	[69]
	Petropoulos et al. (2017)	To evaluate whether CCM detects axonal degeneration and whether this is associated with retinal nerve fiber degeneration and clinical disability in patients with MS.	cross-sectional (50 subjects: 25 MS, 25 controls)	CNFD、CNBD、CNFL were reduced patients with MS compared with healthy controls. The EDSS and MSSS correlated significantly with CNBD.	[70]
Dementia	Ponirakis et al. (2019)	To determine whether there is any association of corneal nerve fiber measures with cognitive function and functional independence in patients with MCI and dementia.	cross-sectional (76 subjects: 30 MCI, 26 dementias, 20 controls)	CNFD、CNBD、CNFL were reduced in patients with MCI and dementia compared to controls. CNFD、CNBD、CNFL were significantly associated with cognitive function and functional independence in MCI and dementia.	[25]
ALS	Ferrari et al. (2014)	To examine a group of sporadic ALS patients with CCM.	cross-sectional (15 subjects: 8 ALS, 7 controls)	CNFD、CNFL were reduced but CNT was increased in ALS patients compared with healthy controls. ALS-SS-bulbar score was significantly related to CNFL and CNFD.	[71]
FRDA	Pagovich et al. (2018)	To evaluated the severity of neurological manifestations in FRDA with CCM.	cross-sectional (37 subjects, 23 FRDA, 14 controls)	CNFD、CNFL were reduced in FRDA compared to healthy controls.	[75]
WD	Sturniolo et al. (2015)	to investigate central corneal changes and in particular to assess the parameters of corneal SBNP in patients affected by WD.	cross-sectional (48 subjects: 24 WD, 24 controls)	NFLD, NF, NBe and NFr were lower, whereas FT was significantly higher in WD subjects compared to controls.	[72]
IS	Khan et al. (2017)	To investigate the use of CCM in patients presenting with acute IS.	cross-sectional (158 subjects: 130 acute IS, 28 controls)	CNFD、CNBD、CNFL were reduced in patients with acute IS compared with healthy controls.	[73]
	Gad et al. (2019)	To determine if CCM can identify corneal nerve and endothelial cell abnormalities with TIA or minor IS.	cross-sectional (54subjects: 14 TIA, 22 minor IS, 18 controls)	CCM identifies corneal nerve fiber loss and endothelial cell abnormalities in patients with TIA and minor IS.	[74]

*MS* Multiple Sclerosis, *DC* dendritic cell, *CNFD* corneal nerve fiber density, *CNBD* corneal nerve branch density, *CNFL* corneal nerve fiber length, *CCM* corneal confocal microscopy, *EDSS* expanded disability status scale, *MSSS* multiple sclerosis severity score, *MCI* mild cognitive impairment, *ALS* amyotrophic lateral sclerosis, *ALS-SS-bulbar score* Amyotrophic lateral sclerosis- Functional Rating Scale-bulbar score, *CNT* corneal nerve tortuosity, *FRDA* Friedreich’s ataxia, *WD* Wilson Disease, *SBNP* sub-basal nerve plexus, *NFLD* nerve fiber length density, *NF* number of fibers, *NBe* number of beadings, *NBr* number of branchings, *FT* fiber tortuosity, *IS* ischemic stroke, *TIA* transient ischemic Attack

### Limitations of CCM

A limitation of CCM is the relatively small field of view, which allows only a proportion of the total sub-basal nerve plexus to be scanned at any given time. Manual image analysis is time-consuming and subjective, while automatic image analysis software is prone to have errors in identifying the nerve trunk and branch nerve fibers. Although there is no standard procedure for CCM image capture, selection and analysis, Kalteniece et al. have proposed a number of steps towards protocol development for image selection and the number of images required for adequate quantification of corneal nerve pathology recently [76]. Following this standardized protocol, selecting and analyzing images of the corneal sub-basal plexus with automated quantification demonstrated excellent inter and intra observer repeatability and concordance, irrespective of investigator experience and image number. In the context of using CCM parameters as a surrogate marker for quantitative detection of peripheral neuropathy, for example longitudinal or intervention trials, especially in multicenter and international cooperation studies, there is considerable variability because of the subjective criteria applied. Using manual image analysis for corneal nerve morphology, Petropoulos et al. demonstrated good intraobserver and interobserver repeatability and consistency between the two eyes for CNFD and CNFL, but relatively lower repeatability for CNBD and CNFT [77]. The authors proposed applying predefined identification rules for the nerve fibers and their branches and deploying fully automated image analysis system to eliminate inconsistencies, enhance repeatability and reduce the analysis time in clinical practice. Also, absence of reference value of CCM parameters for PD patients also limited its clinical application and related study. Although some researchers have provided the cut-off value for CCM [39], its clinical significance still needs to be validated and verified.

## Conclusion

As a new convenient, non-invasive, in vivo ophthalmic imaging technology, CCM can be used to evaluate the damage and repair of small fibers accurately and rapidly in Parkinson’s disease and other neurologic disease. It has prominent advantages in early diagnosis, quantitative analysis of the severity of neural impairment, evaluation and prediction of therapeutic effect in peripheral neuropathy. But at present, the role of CCM in PD early diagnosis and differential diagnosis from other Parkinsonism is still exploratory. There is increasing evidence that peripheral neuropathy in PD is a kind of small fiber neuropathy, a characteristic of neurodegenerative process intrinsic to PD. Since most of CCM study for PD is of small sample size and cross-section character, therefore inconsistent findings between different research groups are common. Thus large-scale, longitudinal, prospective study is urgently needed, and cut-off value for CCM parameters determined before it can be extensively used in clinical practice in PD research.

## Data Availability

Not applicable.

## Abbreviations

CCM: Corneal confocal microscopy
CNBD: Corneal nerve branch density
CNFD: Corneal nerve fiber density
CNFL: Corneal nerve fiber length
CNFT: Corneal nerve fiber tortuosity
IENFD: Intraepidermal nerve fiber density
IW: Inferior whorl
MCI: Mild cognitive impairment
LBD: Lewy body dementia
MSA: Multiple system atrophy
NCS: Neural conduction studies
PD: Parkinson’s disease
PSP: Progressive supranuclear palsy
QST: Quantitative sensory testing
UPDRS: Unified Parkinson’s disease rating scale.
